# 636. *Pneumocystis* Pneumonia Outcomes in Solid Organ Transplant Recipients and Patients Living with HIV: A Population-Based Study Over 20 Years (2002-2022) in Ontario, Canada

**DOI:** 10.1093/ofid/ofae631.201

**Published:** 2025-01-29

**Authors:** Carson Lo, Daniel Fridman, Jeffrey Kwong, Seyed M Hosseini-Moghaddam

**Affiliations:** McMaster University, Mississauga, Ontario, Canada; ICES, Toronto, Ontario, Canada; ICES, Toronto, Ontario, Canada; University Health Network, Toronto, Ontario, Canada

## Abstract

**Background:**

While it is well established that HIV-positive and non-HIV immunocompromised patients are susceptible to *Pneumocystis* pneumonia (PCP), comparing PCP outcomes between patients with different underlying immunocompromising conditions is critically important to optimize chemoprophylaxis strategies.

**Methods:**

In this population-based cohort, we determined contributing factors to 90-day all-cause mortality following PCP diagnosis among patients living with HIV (PLWH) and solid organ transplant recipients (SOTRs) hospitalized in Ontario, Canada between Apr/1/2002 and Dec/31/2022. We linked data from provincial health administrative databases using unique encoded identifiers and included all adult patients hospitalized with PCP based on diagnostic codes from hospital discharge abstracts. We used logistic regression models to estimate unadjusted and adjusted odds ratios (uOR and aOR) and 95% confidence intervals (95%CI) for the associations between underlying immunocompromising conditions and PCP outcomes.

**Results:**

A total of 879 patients were hospitalized with PCP, including 121 SOTRs (13.7%) and 758 PLWH (86.2%). 90-day mortality rates were 19.8% in SOTRs and 13.2% in PLWH. In the unadjusted model, 90-day mortality was not significantly associated with organ transplantation (OR=1.58; 95%CI, 0.96-2.62) or HIV infection (OR=0.62; 95%CI, 0.38-1.03). Comparing HIV patients to liver SOTRs, liver SOTRs experienced higher mortality (uOR=8.54, 95%CI=3.11-23.46). In the adjusted model, liver transplantation was associated with an increased risk of 90-day mortality (aOR=4.36; 95%CI, 1.40-13.59). Among SOTRs, in the unadjusted analysis, liver transplant recipients had an increased risk of 90-day mortality (uOR=9.18; 95%CI, 3.00-28.09).

**Conclusion:**

Among SOTRs, liver transplant recipients are at particularly higher risk of mortality following PCP diagnosis. Our findings may prompt a re-evaluation of current PCP prophylactic strategies in liver transplant patients.

**Disclosures:**

**All Authors**: No reported disclosures

